# Deletion of TRIB3 disrupts the tumor progression induced by integrin αvβ3 in lung cancer

**DOI:** 10.1186/s12885-022-09593-2

**Published:** 2022-04-26

**Authors:** Wen Zhou, Junjun Ma, Lifeng Meng, Dabei Liu, Jun Chen

**Affiliations:** 1 Department of Lung Cancer Surgery, Tianjin Medical University General Hospital, No.154 Anshan Road, Heping District, Tianjin City, 300052 China; 2 Department of Thoracic Surgery, Shanxi Provincial People’s Hospital, Taiyuan, China

**Keywords:** Integrin αvβ3, TRIB3, FAK/AKT, Lung cancer

## Abstract

**Background:**

Integrin αvβ3 has been proposed as crucial determinant for tumor sustained progression and a molecular marker for the estimation of tumor angiogenesis. Our study suggested that integrin αvβ3 could efficiently promote lung cancer cell proliferation and stem-like phenotypes in a tribbles homolog 3 (TRIB3) dependent manner.

**Result:**

Integrin αvβ3 could mediate the activation of FAK/AKT pro-survival signaling pathway. Meanwhile, activated TRIB3 interacted with AKT to upregulated FOXO1 and SOX2 expression, resulting in sustained tumor progression in lung cancer. Our further analysis revealed that TRIB3 was significantly upregulated in lung tumor tissues and correlated with the poor outcome in clinical patients, indicating the potential role of TRIB3 in diagnostic and prognostic estimation for patients with lung cancer.

**Conclusion:**

Our study showed here for the first time that integrin αvβ3 promote lung cancer development by activating the FAK/AKT/SOX2 axis in a TRIB3 dependent signaling pathway, and interrupting TRIB3/AKT interaction significantly improved the outcome of chemotherapy in tumor-bearing mice, representing a promising therapeutic strategy in lung cancer.

**Supplementary Information:**

The online version contains supplementary material available at 10.1186/s12885-022-09593-2.

## Introduction

Lung cancer is the most common malignant carcinoma with a leading cause of cancer associated death worldwide. Despite advance in expounding mechanism of lung carcinogenesis and new surgical/chemotherapeutic protocols, the medium survival time of lung cancer patients remains less than 5 years [1, 2]. Herein, there is an urgent demand to explore the underlying mechanism of lung cancer progression and novel strategies for tumor therapy.

Integrins are dimeric adhesion receptors that is associated with a series of intracellular signals [3]. Interaction between integrins and extracellular matrix could regulate diverse cellular functions, which is strictly correlated with tumor growth and distant metastasis [4]. The alterations in integrin expression level have been extensively reported and are recognized as crucial determinant for neoplastic progression. Compelling reports suggested that the expression of integrin αvβ3 has been detected in various tumor tissues, which strongly suggested the potential role integrin αvβ3 in tumor progression [5]. Indeed, increasing evidence demonstrated that integrin αvβ3 correlated with diverse tumor progression. And inhibition of integrin αvβ3 signaling could strengthen antiangiogenic and antitumor effects of radiotherapy in several tumor types [6, 7]. Also, integrin αvβ3 is capable of facilitating PI3K/AKT signaling pathway activation to promote non-small cell lung cancer cells A549 proliferation [8]. However, the underlying mechanism of integrin αvβ3 induced tumor progression remained poorly understood and the failure of integrin αvβ3 inhibitors for lung cancer treatment in clinical trials indicated the complex mechanism of integrin αvβ3 associated tumor progression.

The pseudokinases TRIBs are functional regulators of cells proliferation and differentiation. TRIBs have been recognized as a stressor in response to cues from tumor microenvironment [9, 10]. Increasing evidence suggested that the expression of TRIBs correlated with cisplatin resistance in lung cancer stem cells [11]. Among TRIB family, TRIB3 have been demonstrated to promote inflammation and cancer development by interacting with intracellular signaling molecules and proteins. And the expression of TRIB3 is strictly correlated with the progression of several tumor types, including breast cancer, colorectal cancer and glioma [12, 13]. Given the crucial role of TRIB3 in a variety of pro-tumor signals, we wondered whether TRIB3 contributed to the pathogenesis of lung cancer and correlated with the prognosis of patients.

In this study, we aimed to further explore the underlying mechanism of integrin αvβ3 induced lung cancer progression. Our findings suggested that integrin αvβ3 could facilitate the FAK/AKT signaling pathway activation in a TRIB3 dependent manner. Interrupting the interaction between TRIB3 and AKT contributed to suppression of lung cancer progression induced by integrin αvβ3. Our study further expounded the underlying mechanism of integrin αvβ3-induced lung cancer progression, which descripting novel indicator for tumor progression, and provided innovative target for lung cancer therapy.

## Materials and methods

### Cell lines and reagents

Human lung cancer cells A549 (established in 1972 by D.J. Giard, et al., through an explant culture of adenocarcinomic lung tissue of a 58-year-old Caucasian male, belonging to hypotriploid alveolar basal epithelial cells) and PC-9 (a human non-small cell lung cancer (adenocarcinoma) with EGFR mutation) were purchased from Cell Bank of Chinese Academy of Sciences (Shanghai, China). All cell lines were cultured in RPMI-1640 (Gibico, MA, USA) supplemented with 10% fetal bovine serum (Gibco, MA, USA). Integrin αvβ3 positive/negative cells were isolated using fluorescence-activated cell sorting. Tumor cells were labeled with 5 μl anti-integrin αvβ3 antibody (ab190147, Abcam, Cambridge, UK) per 10^6^ cells. Integrin αvβ3 positive/negative populations were sorted using a FACSAria machine (BD, CA, USA). FAK inhibitor Y15 and AKT inhibitor 3CAI were purchased from MCM (NJ, USA). Pep2-Ae was purchased from Solarbio (Beijing, China). Cisplatin (Cis) and paclitaxel (PTX) were purchased from Sigma (NJ, USA).

### Cell proliferation analysis

Cell proliferation was determined using the CCK8 kit (Biyuntian, Beijing, China). Briefly, 1 × 10^3^ treated A549 or PC-9 cells were seeded into 96-well culture plates. 20 μl of CCK-8 solution was added into the 96 wells in determined time points. After 37 °C incubation of 2 h, absorbance was measured at 450 nm on a microplate reader (Bio-Rad, MA, USA). Each experiment was performed for independent three times.

### Colony formation

Colony formation assay was conducted to evaluate the tumorigenic potential of cancer cells. Briefly, A549 or PC-9 cells (200 cells per well) were seeded into the 6-well plates and cultured at 37 °C for 14 days. After that, the colonies were fixed by 4% paraformaldehyde and stained by crystal violet. Colonies were pictured and counted. Each experiment was repeated independently in triplicate.

### Transwell analysis

Transwell analysis was conducted to evaluate cell migration of cancer cells. A549 or PC-9 cells (1 × 10^5^ cells) were seeded in the upper transwell chamber (8 μm, Corning, CA, USA). The bottom chamber was filled with 0.5 ml medium containing 20% FBS. After 24 h, cells were fixed with 4% paraformaldehyde, and then stained with 0.05% crystal violet. The cells numbers were count. Each experiment was repeated independently in triplicate.

### Western blotting

Western blotting was performed to examine the protein level of targeted signaling molecule. The protein lysates from A549 and PC-9 cells were separated by SDS-PAGE and then transferred to polyvinylidene fluoride (PVDF) membranes (Millipore, MA, USA). The membrane was incubated with the primary antibodies against to anti-p-FAK (ab81298, 1:1000, Abcam, Cambridge, UK), anti-t-FAK (ab40794, 1:1000, Abcam, Cambridge, UK), anti-p-AKT (ab38449, 1:1000, Abcam, Cambridge, UK), anti-t-AKT (ab8805, 1:1000, Abcam, Cambridge, UK), anti-FOXO1 (ab179450, 1:1000, Abcam, Cambridge, UK), anti-SOX2 (ab92494, 1:1000, Abcam, Cambridge, UK), anti-TRIB3 (ab75846, 1:1000, Abcam, Cambridge, UK) and anti-β-actin (ab8226, 1:1000, Abcam, Cambridge, UK), followed by incubation with an HRP-conjugated secondary antibody (1:1000, Abcam, Cambridge, UK).

### Co-immunoprecipitation (co-IP)

Sorted tumor cells were lysed with coimmunoprecipitation buffer (25 mM Tri-cl (pH 7.4), 150 mM NaCl, 0.5% NP-40, 2.5 mM MgCl, 0.5 mM EDTA, 5% Glycerol). Samples were then incubated with IP antibodies overnight at 4 °C. After that, samples were incubated with Protein A/G Plus-Agarose (Thermo, MA, USA) for 2 h at 4 °C. AKT-TRIB3 interaction complexes were separated from the beads by boiling and subjected to SDS-PAGE, detected using immunoblotting.

### Cytotoxicity analysis

The cytotoxicity of A549 or PC-9 cells to chemotherapy or inhibitor was analyzed using the FITC-Annexin V/ PE-PI apoptosis detection kit (BD, NJ, USA). Briefly, agents treated tumor cells were resuspended and stained with FITC-Annexin V and PE-PI staining solution for 15 min. Then cells apoptosis was detected by flow cytometry on a C6 flow cytometer (BD, NJ, USA). Each experiment was repeated for three independent times.

### RNA interference

For small interfering RNA (siRNA) inhibition of TRIB3, human TRIB3 siRNA (5′-GCGGUUGGAGUUGGAUGACAACUUA-3′ and 5′-GCGUGAUCUCAAGCUGUGUCGCUUU-3′) were obtained from Qingke Co (Beijing, China). A549 and PC-9 cells were transfected with siRNA at a concentration of 20 μmol/ ml using lipofectamine RNAiMAX (Thermo, MA, USA). The TRIB3 silence efficiency was determined using quantified polymerase chain reaction (qPCR) or western blotting.

### Animal protocols

Female NOD-SCID mice (6 ~ 8 weeks) were purchased from Huafukang (Beijing, China). All mice were housed in a specific pathogen-free facility. All animal experiments were performed according to the guidelines approved by the Institute Ethics Committee of Tianjin Medical University General Hospital. To explore the anticancer effects of chemotherapy combining molecule inhibitor, subcutaneous lung cancer model was established. 10^6^ A549 cells (50 μl PBS) were subcutaneously injected into NOD-SCID mice. After two weeks, mice were treated with PBS, PTX (5 mg/kg), Cis (5 mg/kg) and Pep2-Ae (10 mg/kg) by tail vein injection every two days. The tumor volumes were of mice were recorded every day (*n* = 6). Survival was recorded on a daily basis (*n* = 6). The calculation formula of tumor volume: tumor volume = length × width ^2^/2. For tumorigenesis analysis, 10^5^ A549 cells (50 μl PBS) or PC-3 cells (50 μl PBS) were subcutaneously injected into NOD-SCID mice. After 30 days, the tumor-bearing mice were counted. Each experiment was repeated independently in triplicate.

### Statistical analysis

The TCGA data were downloaded from http://ualcan.path.uab.edu/index.html and https://www.cbioportal.org/. Each experiment was performed for at least three independent times. Results were presented as the mean ± SEM and the statistical significance was analyzed using GraphPad 6.0 software (La Jolla, CA, USA). Statistical significance between groups was calculated by Student’s t test for two groups or by one-way ANOVA for more than two groups. The survival rates were determined by Kaplan–Meier survival analysis, *p < 0.05; **p < 0.01; ns, no significant difference.

## Results

### Integrin αvβ3 promoted tumor progression of NSCLC in vitro

**Fig. 1:**
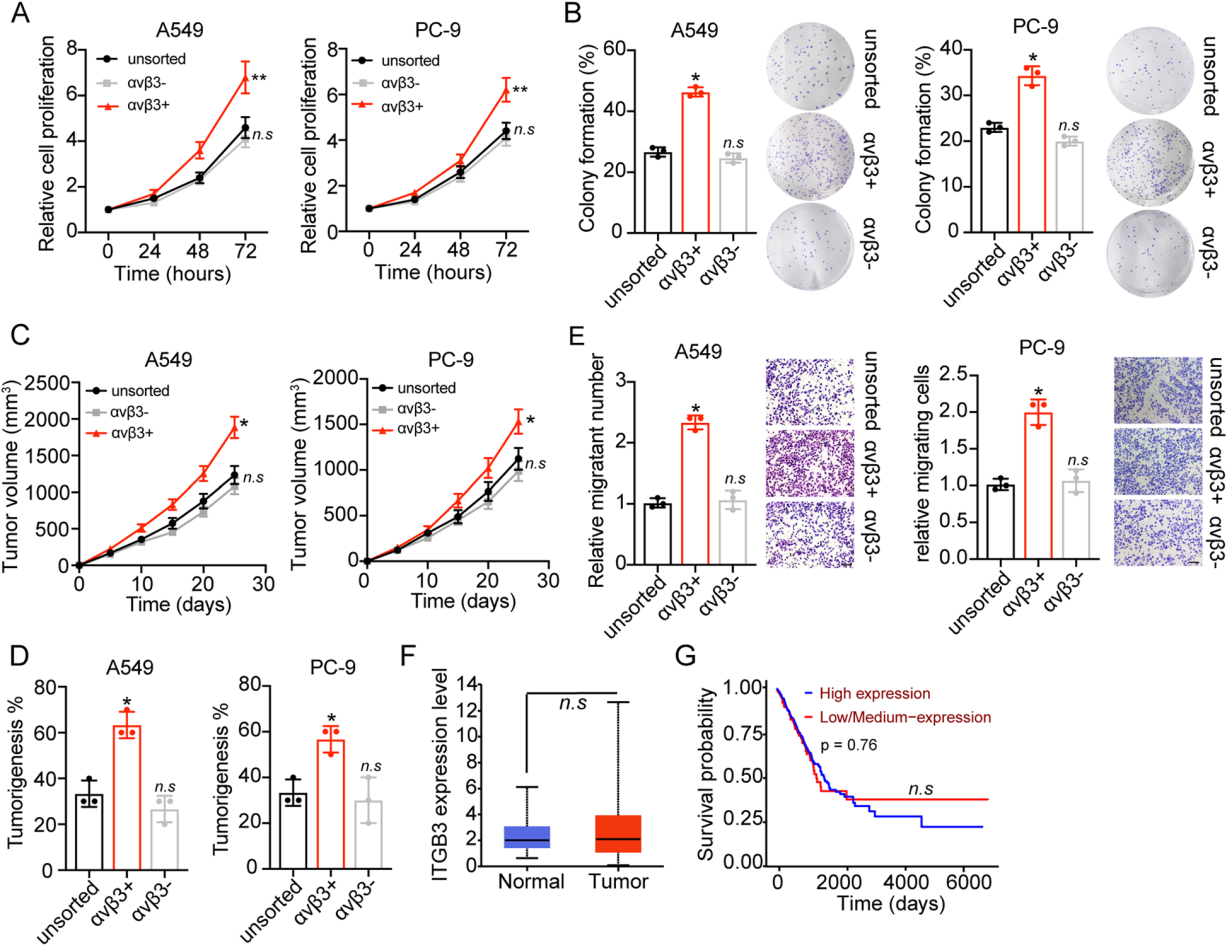
Integrin αvβ3 promoted NSCLC progression in vitro. **A**, relative cell proliferation of A549/PC-9, integrin αvβ3 negative A549/PC-9 and integrin αvβ3 positive A549/PC-9 cells. **B**, the colony formation rates of A549/PC-9, integrin αvβ3 negative A549/PC-9 and integrin αvβ3 positive A549/PC-9 cells. **C**, the tumor volumes of A549, integrin αvβ3 negative A549 and integrin αvβ3 positive A549 bearing mice. **D**, the tumorigenesis percentages of A549, integrin αvβ3 negative A549 and integrin αvβ3 positive A549 cells in immunodeficient mice. **E**, relative migrating cell numbers of A549/PC-9, integrin αvβ3 negative A549/PC-9 and integrin αvβ3 positive A549/PC-9 cells. The scale bar is 15 μm. **F**, the expression of integrin αvβ3 in para-carcinoma tissues (*n* = 59) and tumor tissues (*n* = 515) from NSCLC patients. **G**, the overall survival of NSCLC patients divided into high integrin αvβ3 expression group (*n* = 127) and low integrin αvβ3 expression (*n* = 375). n.s means no significant difference, * means *p* < 0.05, ** means *p* < 0.01

As reported in previous studies, cancer cells with aberrant integrin expression exhibited enhanced stem-like phenotypes and migratory properties [14]. Our aim was to elucidate the potential role of integrin αvβ3 in driving NSCLC progression. To do this, we used fluorescence-activated cell sorting to isolated integrin αvβ3 positive cells from NSCLC cell lines A549 and PC-9. The cell proliferation and colony formation were examined ex vivo, and enhanced capability of proliferation (Fig. 1A) or colony formation (Fig. 1B) was found in αvβ3 positive cells compared to unsorted or αvβ3 negative cells. In consistent, αvβ3 positive A549 cells also revealed strengthened tumor growth (Fig. 1C) and tumorigenesis (Fig. 1D) in immunodeficient mice, indicating that integrin αvβ3 promoted cells proliferation and stem-like phenotypes in NSCLC cells. Tumor cells with stem-like phenotypes frequently showed migratory and invasive properties. Herein, to assess the influence of integrin αvβ3 in cell migration, transwell analysis were conducted in the A549 and PC-9 cells. As a result, integrin αvβ3 significantly promoted A549 and PC-9 cells migration (Fig. 1E). We next explored the role of integrin αvβ3 in NSCLC patient prognosis. However, no significantly difference of integrin αvβ3 expression was observed in the tumor tissues or para-carcinoma tissues (Fig. 1F). And patients with low αvβ3 expression possessed no advantages in overall survival compared with the high αvβ3 expression group (Fig. 1G). Those results suggested that integrin αvβ3 promoted tumor progression of NSCLC in vitro, whereas no positive correlation between integrin αvβ3 and NSCLC patients prognosis was found.

### Integrin αvβ3 mediated FAK/AKT signals to promote NSCLC progression

**Fig. 2:**
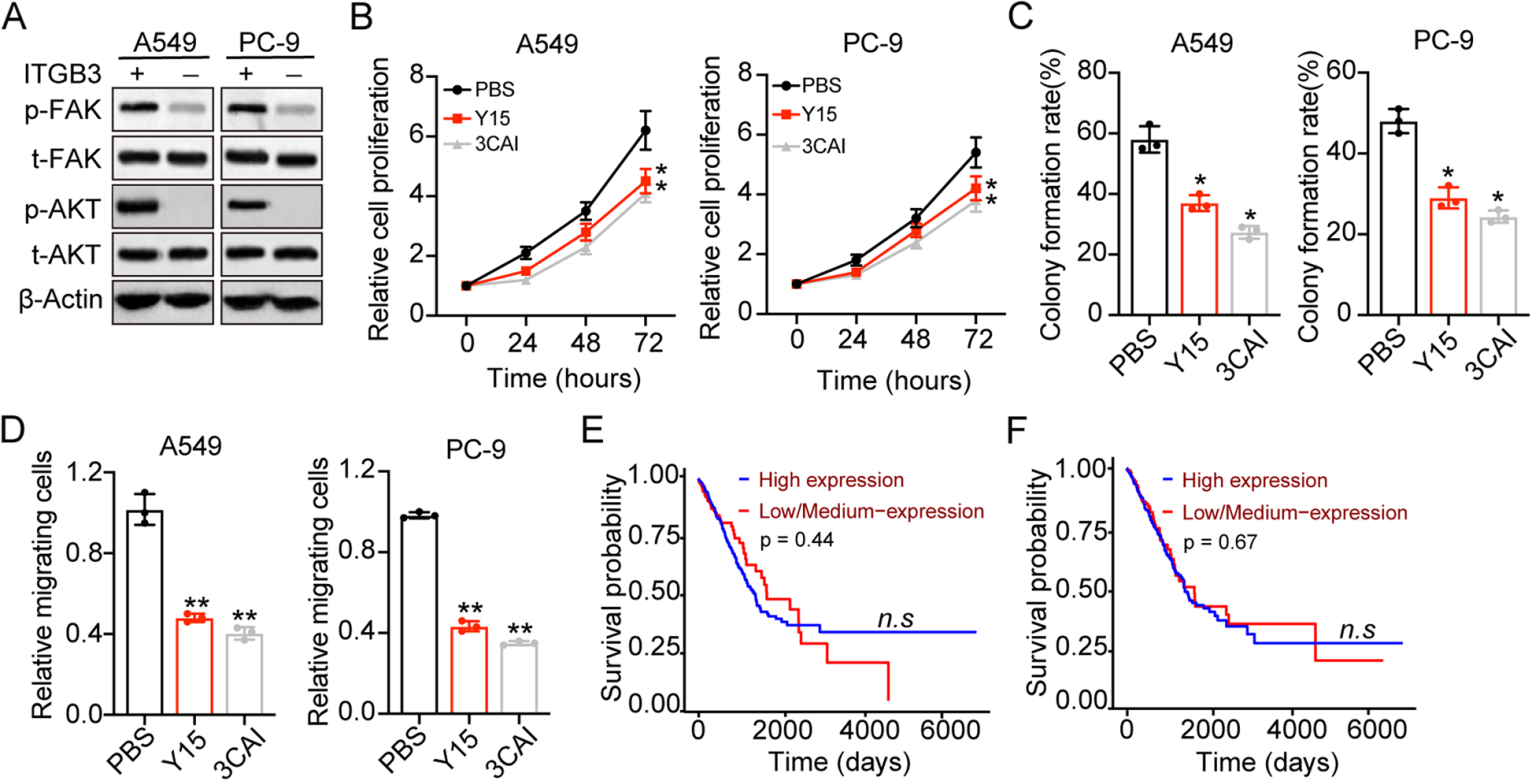
Integrin αvβ3 mediated FAK/AKT signals to promote NSCLC progression. **A**, western blotting of phosphorylated FAK, total FAK, phosphorylated AKT and total AKT in integrin αvβ3 negative A549/PC-9 and integrin αvβ3 positive A549/PC-9 cells. **B**, relative proliferation of αvβ3 positive A549/PC-9 cells treated with PBS, Y15 (20 nM) and 3CAI (10 nM). **C**, the colony formation rates of αvβ3 positive A549/PC-9 cells treated with PBS, Y15 (20 nM) and 3CAI (10 nM). **D**, relative migrating cell numbers of αvβ3 positive A549/PC-9 cells treated with PBS, Y15 (20 nM) and 3CAI (10 nM). **E**, the overall survival of NSCLC patients divided into high FAK expression group (*n* = 127) and low FAK expression (*n* = 375). **F**, the overall survival of NSCLC patients divided into high AKT expression group (*n* = 127) and low AKT expression (*n* = 375). n.s means no significant difference, * means *p* < 0.05, ** means *p* < 0.01

Given that integrin αvβ3 have no influence on NSCLC prognosis, whereas exhibiting pro-tumor effects in vitro, we sought to explore the underlying mechanism of tumor progression induced by integrin αvβ3. Activation of integrins downstream signaling pathways, such as AKT signaling pathway for promoting cell survival, was dependent on the phosphorylation of FAK [15]. Here, the expression of FAK and AKT was examined in A549 and PC-3 cells. Both phosphorylated FAK and phosphorylated AKT were upregulated in αvβ3 positive A549/PC-9 cells (Fig. 2A). We next further suppressed the FAK/AKT signals in NSCLC cells by using FAK inhibitor Y15 and AKT inhibitor 3CAI to treat integrin αvβ3 positive A549/PC-9 cells. Intriguingly, blockade of FAK/AKT signals efficiently suppressed the cells proliferation (Fig. 2B) and colony formation (Fig. 2C) induced by integrin αvβ3. Meanwhile, integrin αvβ3 positive A549/PC-9 cells revealed weakened capability of migration after Y15 and 3CAI treatment (Fig. 2D), suggesting that integrin αvβ3 promoted NSCLC cells proliferation through FAK/AKT signaling pathway. Subsequently, we continued to evaluate the influence of FAK/AKT on prognosis of NSCLC patients. However, no direct correlation was observed between FAK/AKT expression and overall survival in NSCLC patients (Fig. 2E and F). Those results suggested that additional signaling molecular might participate in the FAK/AKT associated NSCLC progression.

### Integrin αvβ3 induced tumor progression was TRIB3 dependent

**Fig. 3:**
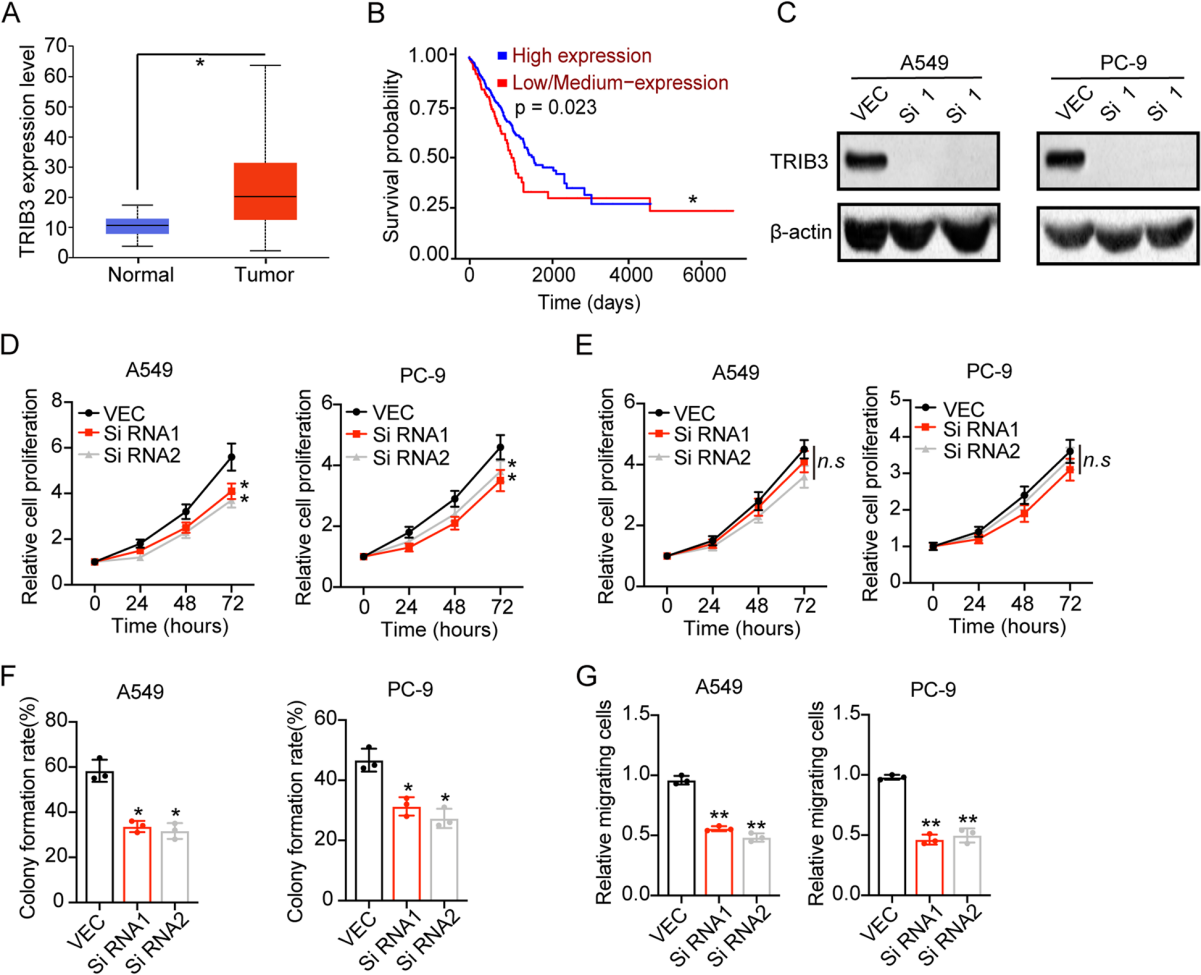
Integrin αvβ3 induced tumor progression was TRIB3 dependent. **A**, the expression of TRIB3 in para-carcinoma tissues (*n* = 59) and tumor tissues (*n* = 515) from NSCLC patients. **B**, the overall survival of NSCLC patients divided into high TRIB expression group (*n* = 127) and low TRIB expression (*n* = 375). **C**, western blotting of TRIB3 in vector control, TRIB3 silenced #1 and TRIB3 silenced A549/PC-9. **D**, relative cell proliferation of vector control, TRIB3 silenced #1 and TRIB3 silenced A549/PC-9 (integrin αvβ3 positive). **E**, relative cell proliferation of vector control, TRIB3 silenced #1 and TRIB3 silenced A549/PC-9 (integrin αvβ3 negative). **F**, the colony formation rates of vector control, TRIB3 silenced #1 and TRIB3 silenced A549/PC-9 (integrin αvβ3 positive). **G**, relative migrating cell numbers of vector control, TRIB3 silenced #1 and TRIB3 silenced A549/PC-9 (integrin αvβ3 positive). * means *p* < 0.05, ** means *p* < 0.01

Compelling findings provided evidence that TRIB3 linked stress signals are capable of promoting tumor initiation and progression by supporting cancer stemness, which is coordinated with elevated FOXO1 and AKT1 axis [16]. Herein, we analyzed TRIB3 expression and overall survival in NSCLC patients from TCGA database. Intriguingly, elevated expression of TRIB3 was observed in tumor tissues in comparison with the para-carcinoma tissues (Fig. 3A). And a high correlation was found in TRIB expression and overall survival in NSCLC patients (Fig. 3B), indicating that TRIB3 served as the major regulator in NSCLC progression. Thus, we silenced TRIB3 in A549 and PC-9 cells (Fig. 3C), and further sorted integrin αvβ3 positive NSCLC cells to examine the cell proliferation. Notably, suppression of TRIB3 retarded the proliferative effects induced by integrin αvβ3 in A549/PC-9 cells (Fig. 3D), whereas no significant difference was found in integrin αvβ3 negative NSCLC cells (Fig. 3E). Also, the strengthened capability of colony formation (Fig. 3F) and cell migration (Fig. 3G) was aborted when silencing TRIB3 in αvβ3 positive A549 and PC-9 cells. Collectively, those results suggested that integrin αvβ3 induced tumor progression was TRIB3 dependent.

### TRIB3 interacted with AKT1 to up-regulate FOXO1 and SOX2 expression

**Fig. 4:**
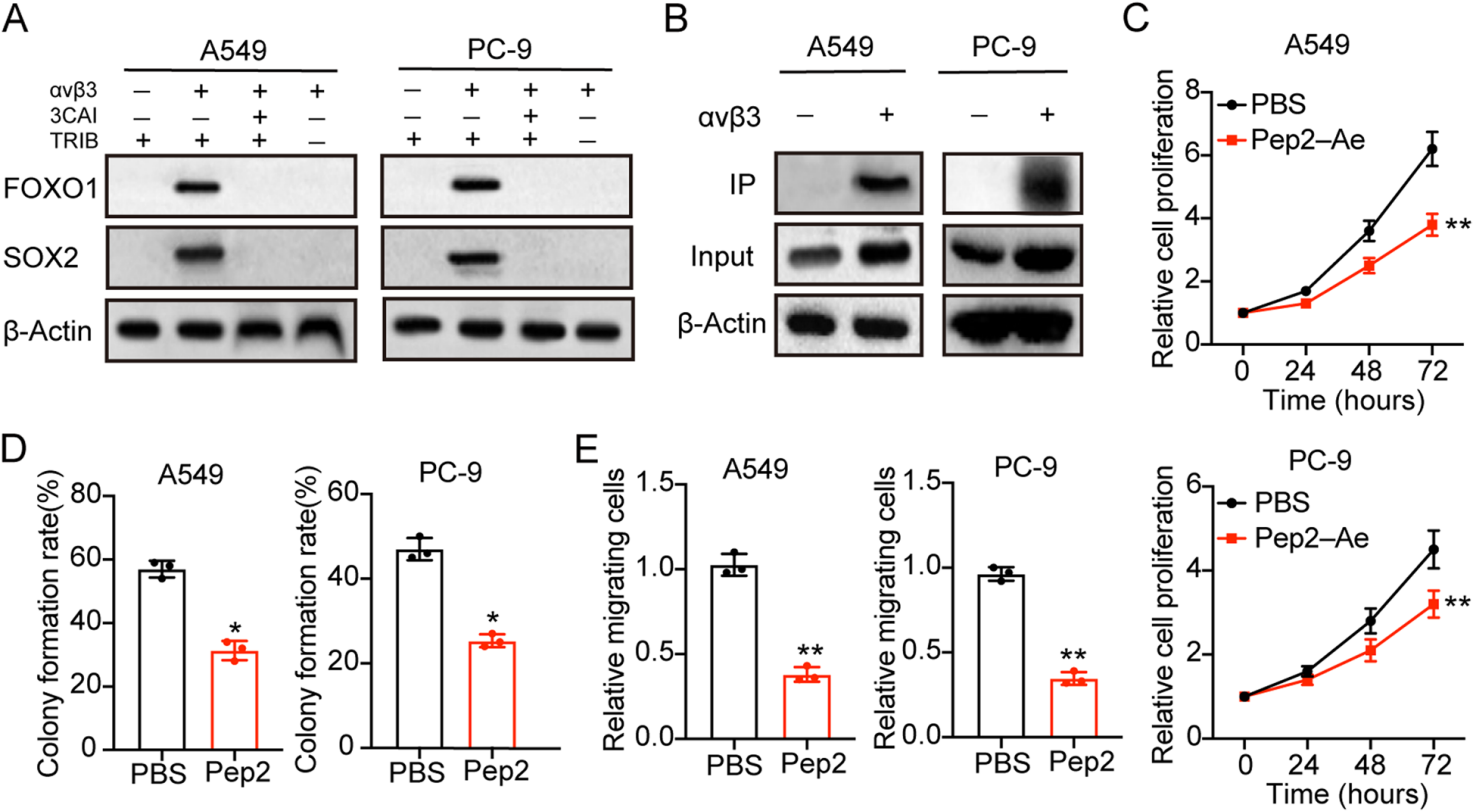
TRIB3 interacted with AKT1 to up-regulate FOXO1 and SOX2 expression. **A**, western blotting of FOXO1 and SOX2 in integrin αvβ3 negative A549/PC-9, integrin αvβ3 positive A549/PC-9 treated with PBS, 3CAI (10 nM) and TRIB3 knockdown. **B**, co-immunoprecipitation of AKT and TRIB3 in integrin αvβ3 negative A549/PC-9, integrin αvβ3 positive A549/PC-9 cells. **C**, relative cell proliferation of integrin αvβ3 positive A549/PC-9 cells treated with PBS or Pep2–Ae (20 μg/ml). **D**, colony formation rates of integrin αvβ3 positive A549/PC-9 cells treated with PBS or Pep2–Ae (20 μg/ml). **E**, relative migrating cell numbers of integrin αvβ3 positive A549/PC-9 cells treated with PBS or Pep2–Ae (20 μg/ml). * means *p* < 0.05, ** means *p* < 0.01

Previous documents have proved that TRIB3 could interact with AKT1 to abrogate FOXO1 degradation, resulting in the up-regulating of FOXO1 and downstream transcription factor SOX2 activation [12]. Firstly, we examined the expression FOXO1 and SOX2 in A549 and PC-3 cells. Notably, enhanced expression of FOXO1 and SOX2 was found in integrin αvβ3 positive A549 and PC-9, and suppression of AKT or TRIB3 retarded up-regulation of FOXO1/SOX2 (Fig. 4A). Next, we wondered whether TRIB3 mediated FOXO1 upregulation by interacting with AKT1. The endogenous AKT1 was co-immunoprecipitated with TRIB3. And obvious interaction between AKT1 and TRIB3 was found in integrin αvβ3 positive A549 and PC-9 cells (Fig. 4B). To further confirm the role of TRIB3-AKT1 axis in NSCLC progression, we used Pep2–Ae to interrupt the contact between TRIB3 and AKT1 and examined the cell proliferation of integrin αvβ3 positive cells. Intriguingly, Pep2–Ae treatment significantly suppressed the cell proliferation (Fig. 4C) and colony formation (Fig. 4D) of integrin αvβ3 positive A549 and PC-9 cells. Meanwhile, interruption of TRIB3-AKT1 axis retarded the cell migration in NSCLC cells (Fig. 4E). Together, those results suggested that TRIB3 contacted with AKT1 and upregulated FOXO1 expression, resulting in the SOX2 activation and NSCLC progression.

### Disruption of the TRIB3-AKT interaction suppressed NSCLC progression

**Fig. 5:**
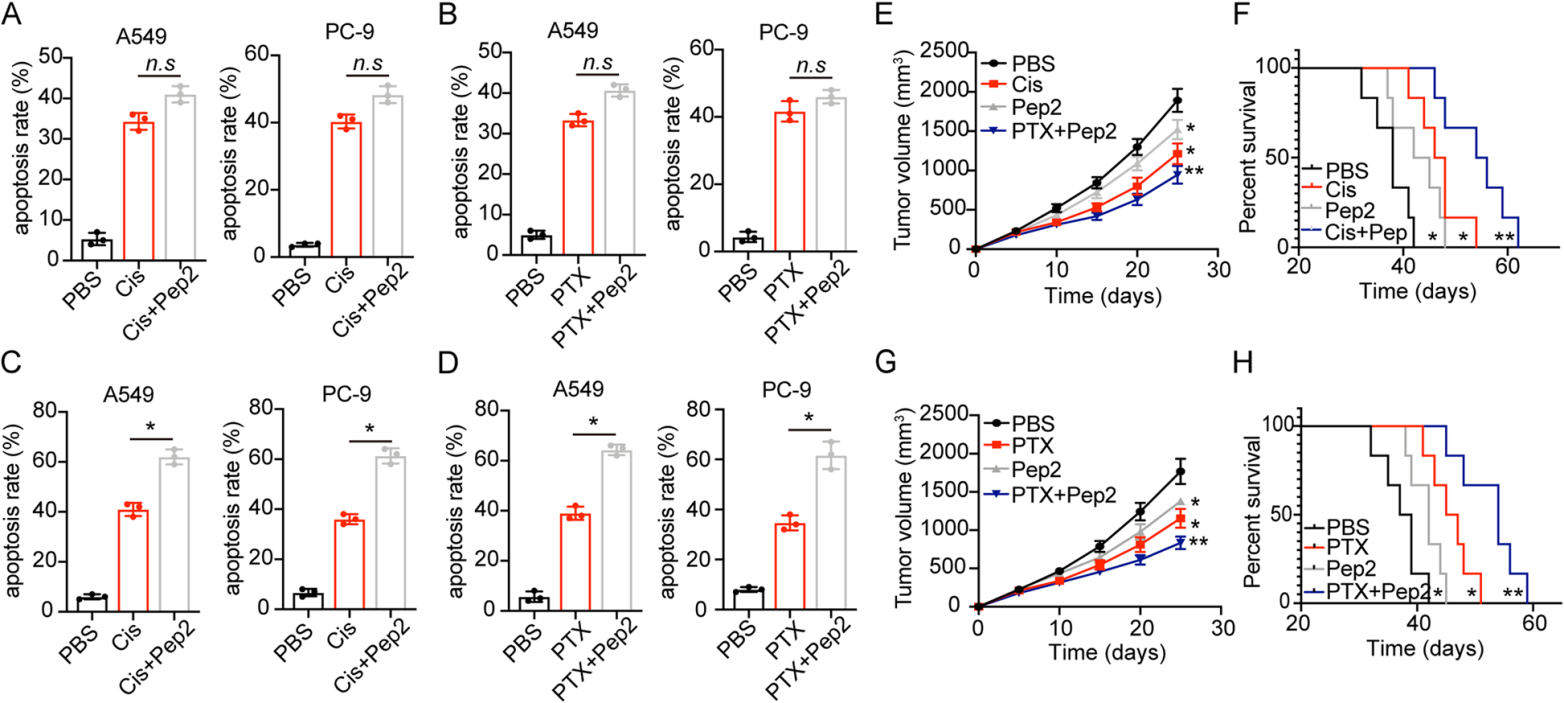
Disruption of the TRIB3-AKT interaction suppressed NSCLC progression. **A**, apoptosis of integrin αvβ3 negative A549/PC-9 treated with PBS, Pep2–Ae (20 μg/ml), Cis (1 μg/ml) and combination. **B**, apoptosis of integrin αvβ3 negative A549/PC-9 treated with PBS, Pep2–Ae (20 μg/ml), PTX (2 μg/ml) and combination. **C**, apoptosis of integrin αvβ3 positive A549/PC-9 treated with PBS, Pep2–Ae (20 μg/ml), Cis (1 μg/ml) and combination. **D**, apoptosis of integrin αvβ3 positive A549/PC-9 treated with PBS, Pep2–Ae (20 μg/ml), PTX (2 μg/ml) and combination. **E**, the tumor volumes of A549 bearing mice treated with PBS Pep2–Ae, Cis and combination. **F**, the overall survival of A549 bearing mice treated with PBS Pep2–Ae, Cis and combination. **G**, the tumor volumes of A549 bearing mice treated with PBS Pep2–Ae, PTX and combination. **H**, the overall survival of A549 bearing mice treated with PBS Pep2–Ae, PTX and combination. n.s means no significant difference, * means *p* < 0.05, ** means *p* < 0.01

Given the importance of TRIB3-AKT axis in NSCLC development, we examined whether disruption of TRIB3-AKT interaction could improve the anticancer effects in NSCLC. Firstly, we combined Pep2–Ae with chemotherapeutic PTX/Cis to treat A549 and PC-9. Intriguingly, Pep2-Ae treatment revealed no influence on the integrin αvβ3 negative cells (Fig. 5A and B), however, significantly strengthened the cytotoxicity of PTX (Fig. 5C) and Cis (Fig. 5D) to integrin αvβ3 positive A549 and PC-9. Those results implicated that blockade of TRIB3-AKT interaction efficiently suppressed the tumor progression induced by integrin αvβ3 and improved the outcome of chemotherapy in NSCLC. Next, tumors arising from the subcutaneous implication of A549 cells in mice was established for anticancer effects analysis in vivo. Using this model, subcutaneous A549 tumors were established in mice and, then treated with chemotherapeutic Cis, PTX and Pep2–Ae. Notably, Pep2–Ae treatment resulted in the suppression of tumor growth, and strengthened the anticancer effects of Cis (Fig. 5E and F) and PTX (Fig. 5G and H) in A549 bearing mice. On the basis of our results, we suggested that suppression of TRIB3-AKT axis could efficiently impair tumor growth and improve the outcome of chemotherapy in NSCLC.

### Discussion

**Fig. 6:**
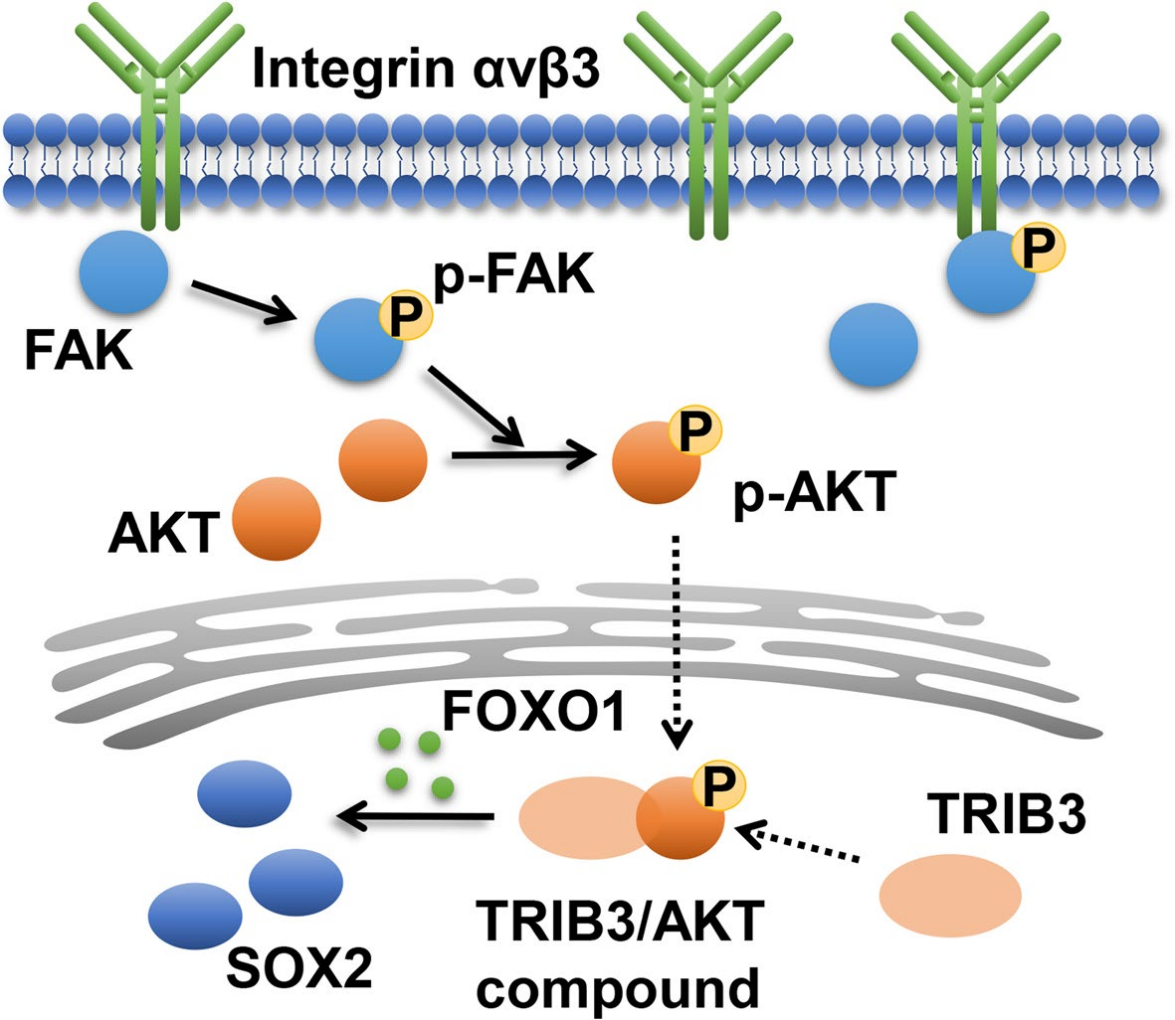
Schematic diagram of integrin αvβ3-TRIB3 induced lung cancer progression

Previous in vitro and in vivo studies have suggested the association between integrin αvβ3 and tumor progression [6, 17]. Our observation that integrin αvβ3 could efficiently promote lung cancer cells progression in vitro, which is in consistent with previous reports. However, the TCGA database analysis implicated that no significant difference was found between the patients with high/low expression of integrin αvβ3. Here, we reported in the present study that integrin αvβ3 facilitated the lung cancer proliferation and invasion through FAK/AKT signaling pathway, which was dependent on the collaboration with TRIB3. Depletion of TRIB3 resulted in the inactivation of AKT signals, the downstream pro-survival signals of integrin αvβ3 (Fig. 6). To our knowledge, these data firstly provided evidence that tumor integrin αvβ3 contributed to the tumor progression through an TRIB3 dependent manner.

Increasing evidence suggested that differential expression cancer stem cell markers, such as ALDH1, CD133 and CD44, was tightly correlated with the pathological subtypes and tumor development of lung cancer [18, 19]. Importantly, there is recent evidence that integrins also potentiate cancer stem cell function, especially in lung cancer. In agreement with previous reports that integrin αvβ3 correlated with the metastasis of lung cancer cells [20], we demonstrated that integrin αvβ3 efficiently promoted the cells invasion in lung cancer. Furthermore, we provided evidence that integrin αvβ3 promoted the stem-like phenotypes and proliferation of A549 and PC-9. Accordingly, compelling findings suggested that integrin αvβ3 served as novel cancer stem cells marker, that contributed the stemness up-regulation in melanoma and breast cancer [21, 22].

In mechanism, C Chetty and his colleagues suggested that MMP-2 could up-regulate VEGF expression via integrin αvβ3/PI3K/AKT signaling in lung cancer cells [8]. And previous reported indicated the activation of integrin αvβ3 and FAK/NF-kB signals was correlated with the cancer metastasis [23]. Our study further demonstrated integrin αvβ3 mediated FAK/AKT signaling pathway activation to promote lung cancer cells proliferation. More importantly, we further demonstrated that TRIB3 interacted with AKT1 to up-regulate FOXO1 and SOX2 expression, which was indispensable for the pro-tumor effects induced by integrin αvβ3.

Overexpression of pluripotency genes, including c-Myc, SOX2 and Oct3/4, could promote the cancer stem cells and contribute to the tumor progression [24–26]. Additionally, clusters of mutations occurring at certain regions of the genome are a previously unrecognized key player in regulating tumor progression [27]. In our study, we demonstrated the aberrant upregulation of TRIB3 correlated with the lung cancer progression, which was associated with the SOX2 transcription factor activation. SOX2 played a crucial role, by not only promoting pluripotency but also facilitating self-renewal and differentiation [28]. SOX2 was overexpressed in several tumor types, including lung and prostate cancer [29, 30]. However, the underlying mechanism by which SOX2 was upregulated in tumor tissues remained unclear. Compelling reports have suggested that specific cellular receptors, such as integrins and EGFR could mediate the pro-survival signaling pathways and SOX2 activation [31]. However, increasing evidence implicated that blockade of upstream integrins receptors by inhibitors was insufficient to suppress the tumor progression induced by those pro-survival signals [32]. More importantly, the expression of integrin αvβ3 was found in abundant tumor cells/tissues, whereas the expression of SOX2 was only found in those cancer stem cells, which accounted for a little proportion in tumor cells [33, 34]. In our study, no significant correlation was found in integrin αvβ3 expression and overall survival in lung cancer patients, despite the pro-tumor effects of integrin αvβ3 in vitro. We demonstrated that the pro-tumor effects and the activation of SOX2 was dependent on a TRIB3 manner, and the patient prognosis was influence by the TRIB3 expression. In mechanism, the interaction between AKT and TRIB3 was necessary for the FOXO1/AKT/SOX2 activation in lung cancer. Our results further described the underlying mechanism of integrin αvβ3 and SOX2 in regulating lung cancer development, which provided new sight for target therapy in lung cancer.

In conclusion, our study suggested that TRIB3 links integrin αvβ3 signals to induce lung cancer progression, which was coordinated with AKT/SOX2 signals. Interrupt of TRIB3/AKT axis by Pep2–Ae efficiently improved the outcome of chemotherapy, which describing an innovative approach for lung cancer treatment.

## Supplementary Information

**Additional file 1**

## Data Availability

The original raw data, including colony formation, transwell experiment analysis, western blotting and related analysis in TCGA cohort conducted in this manuscript, have been uploaded in the website of “https://figshare.com/s/b8745acccee577f2f07b”. The raw data of western blotting could be found in supplementary materials.
