# Supplementary material for: Deletion of TRIB3 disrupts the tumor progression induced by integrin αvβ3 in lung cancer
Source: BMC Cancer. 2022 Apr 26;22:459. doi: 10.1186/s12885-022-09593-2 (PMC9044834; doi:10.1186/s12885-022-09593-2)
Supplement: Supplementary file 1 — Additional file 1 [file 12885_2022_9593_MOESM1_ESM.docx]

WB summary


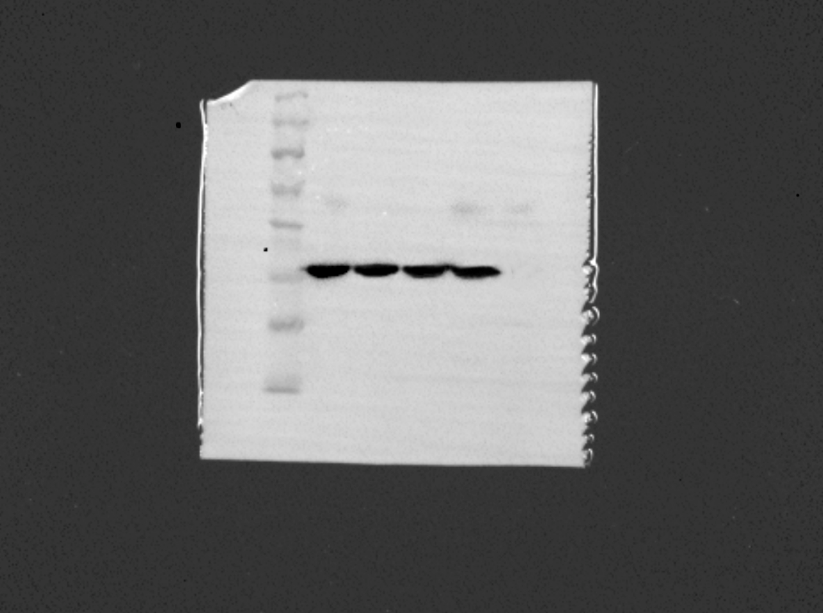


Fig. 2A actin (left A549, right PC-9)


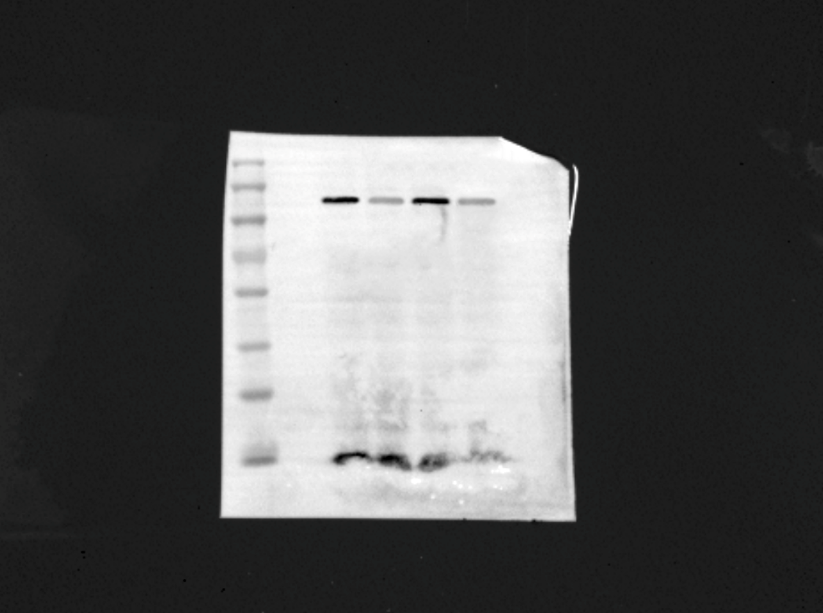


Fig. 2A p-FAK (left A549, right PC-9)


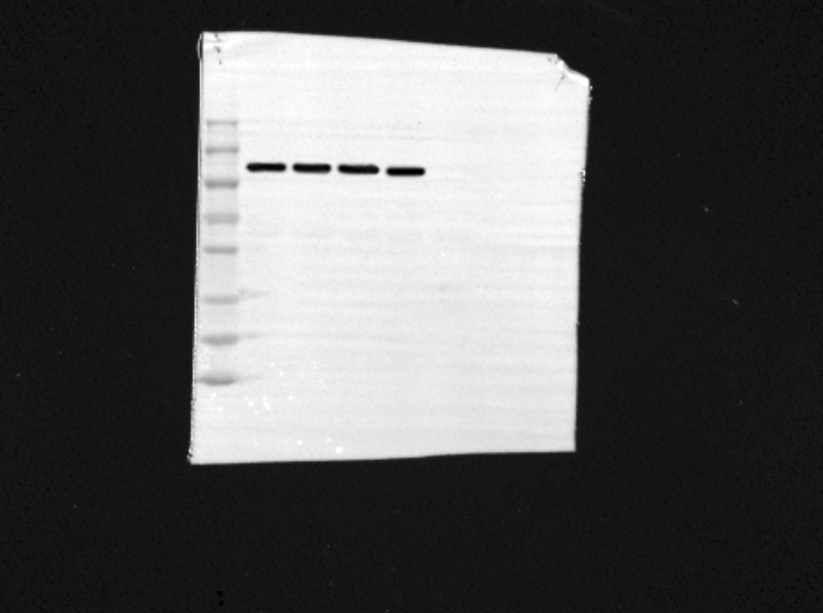


Fig. 2A Total FAK (left A549, right PC-9)


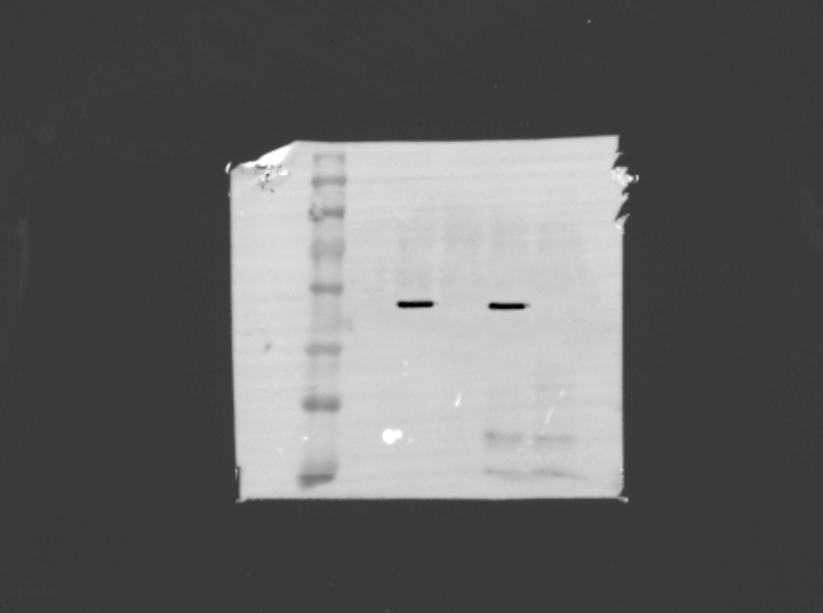


Fig. 2A p-AKT (left A549, right PC-9)


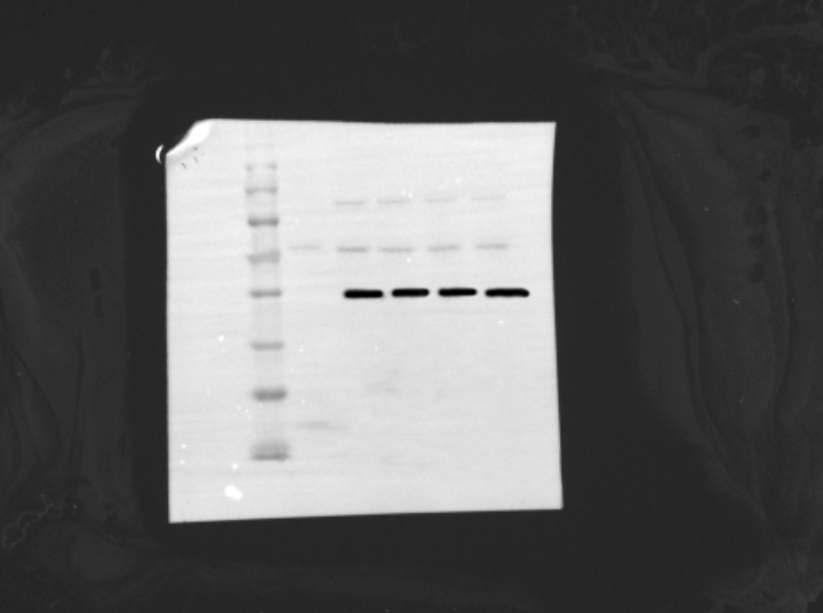


Fig. 2A t-AKT (left A549, right PC-9
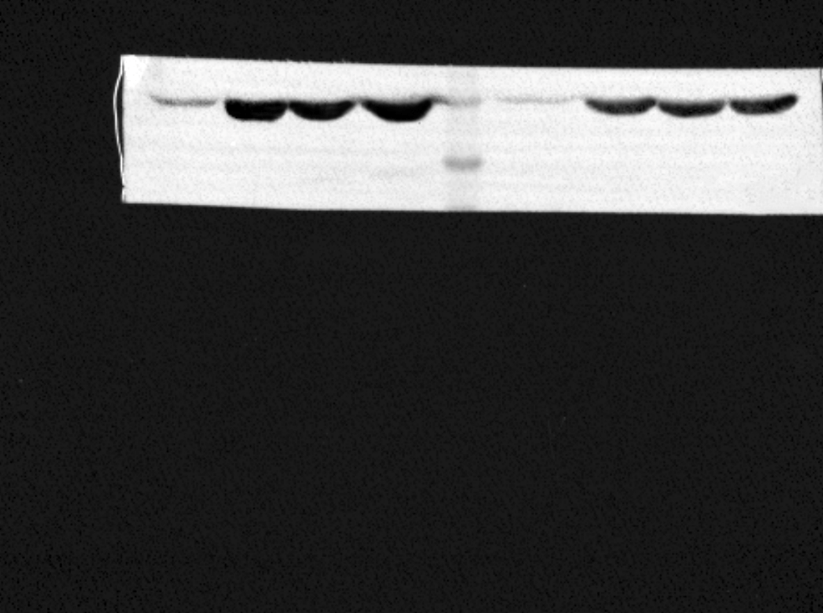
)

Fig. 3C F-actin (left A549, right PC-9)


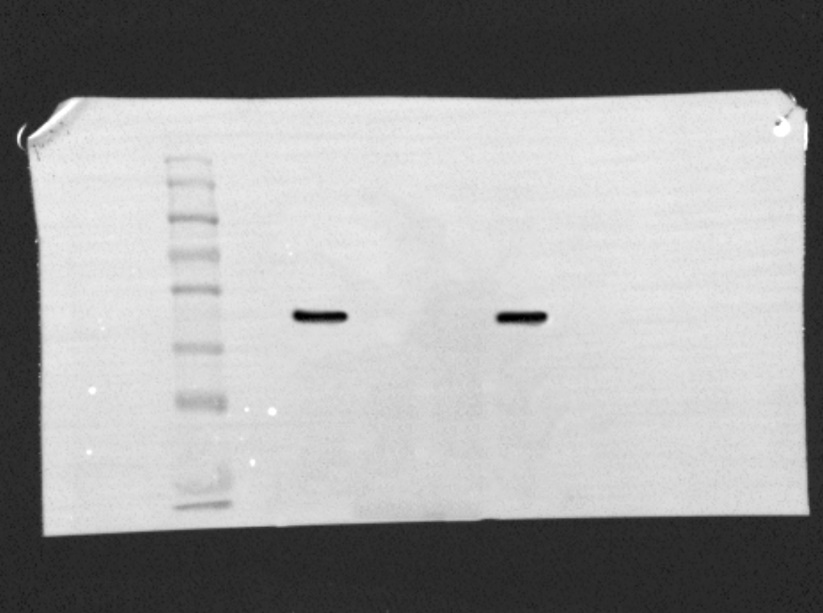


Fig. 3C TRIB3 (left A549, right PC-9)


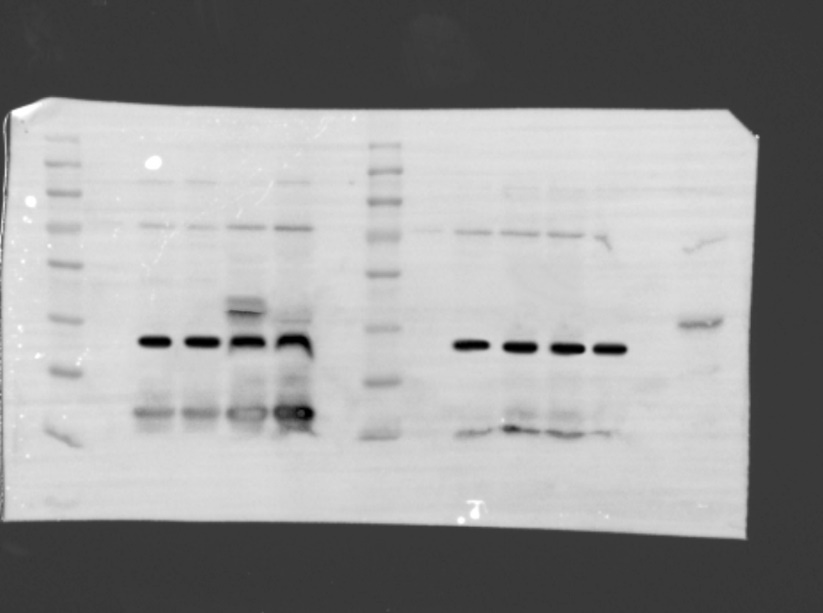


Fig. 4A actin (left A549, right PC-9)


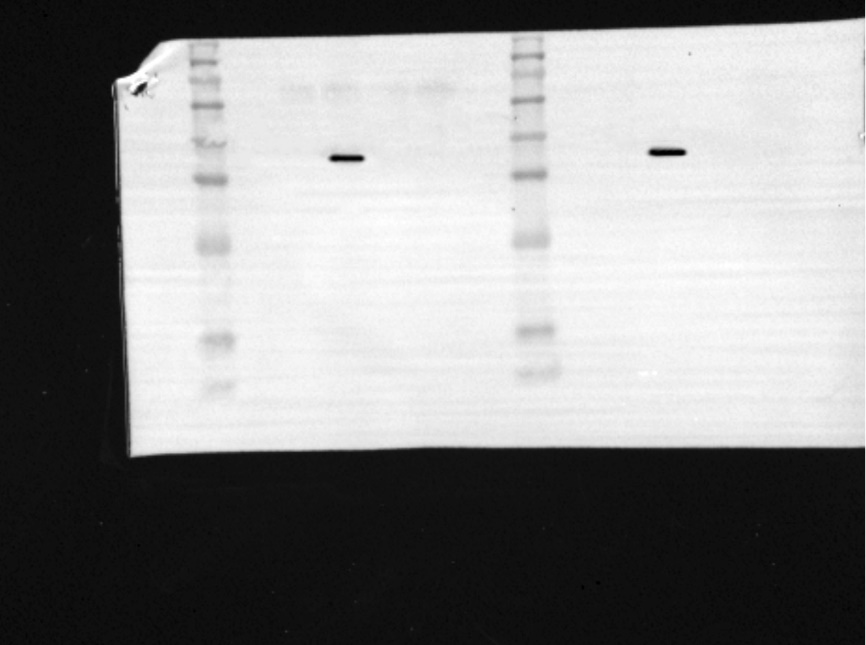
\

Fig. 4A FOXO1 (left A549, right PC-9)


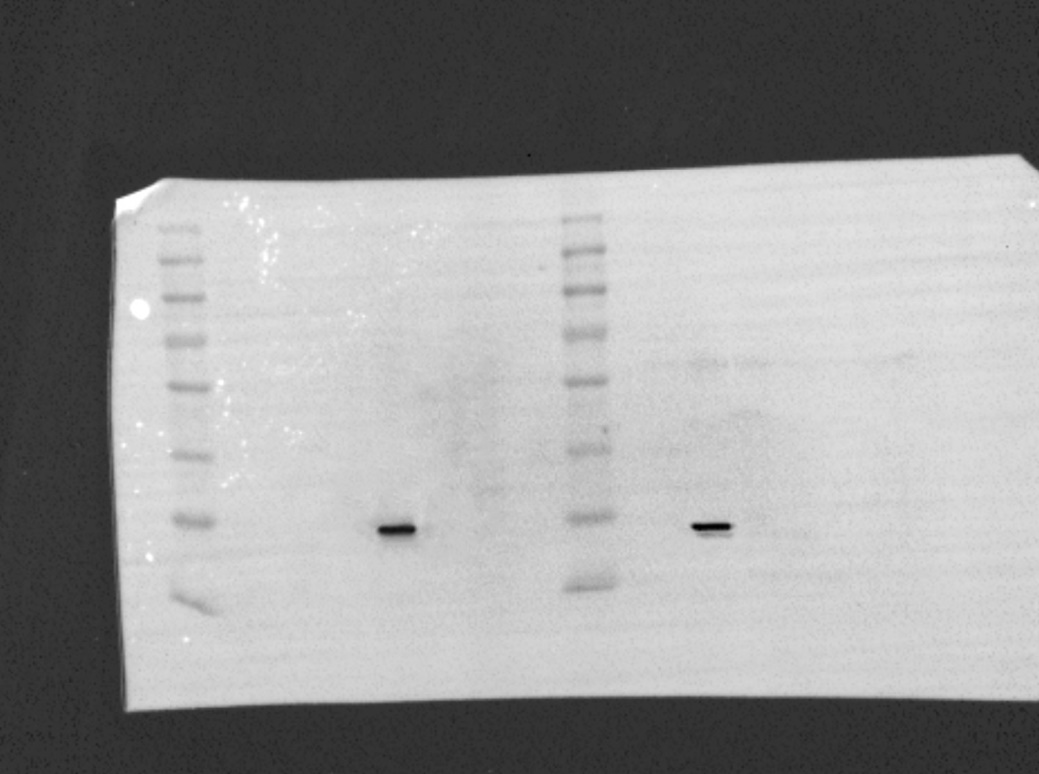


Fig. 4A SOX2 (left A549, right PC-9)


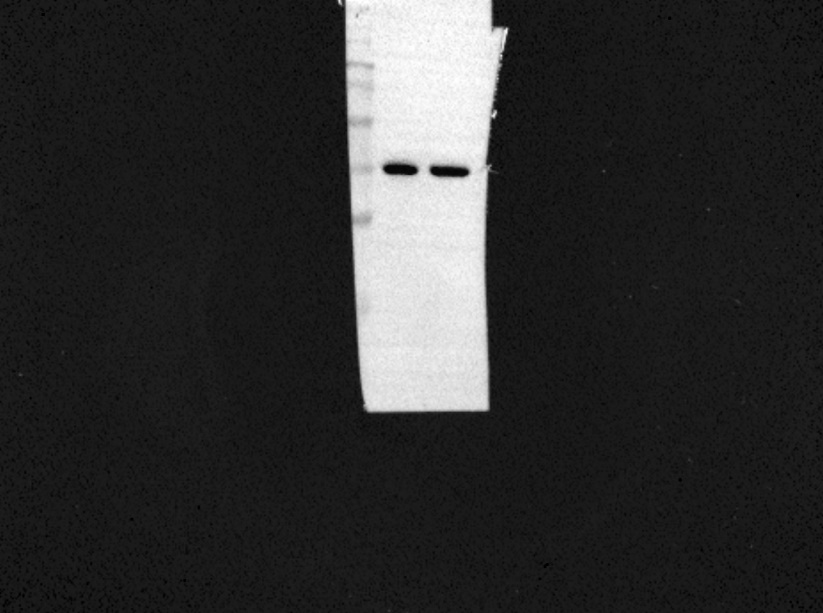


Fig. 4B actin A549


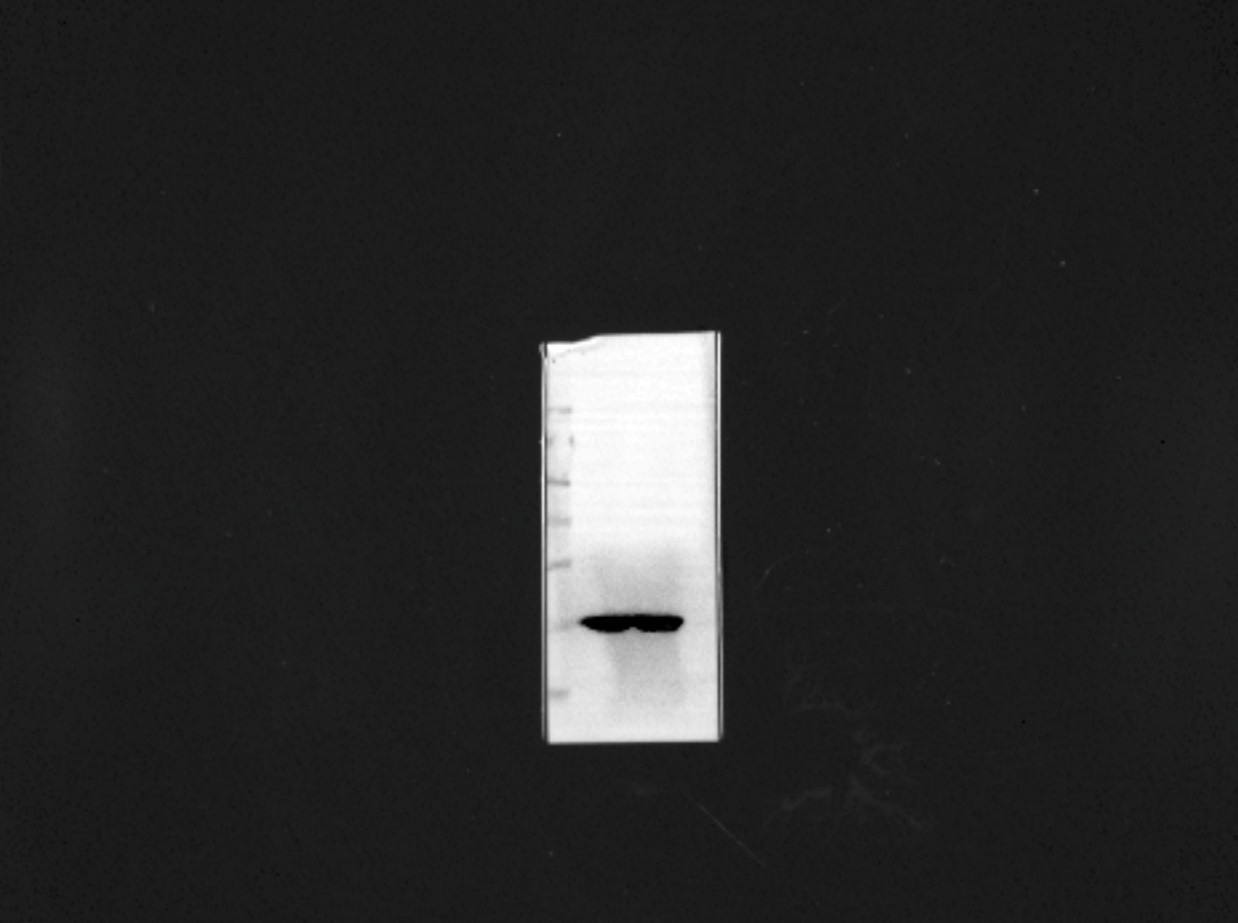


Fig. 4B actin PC-9


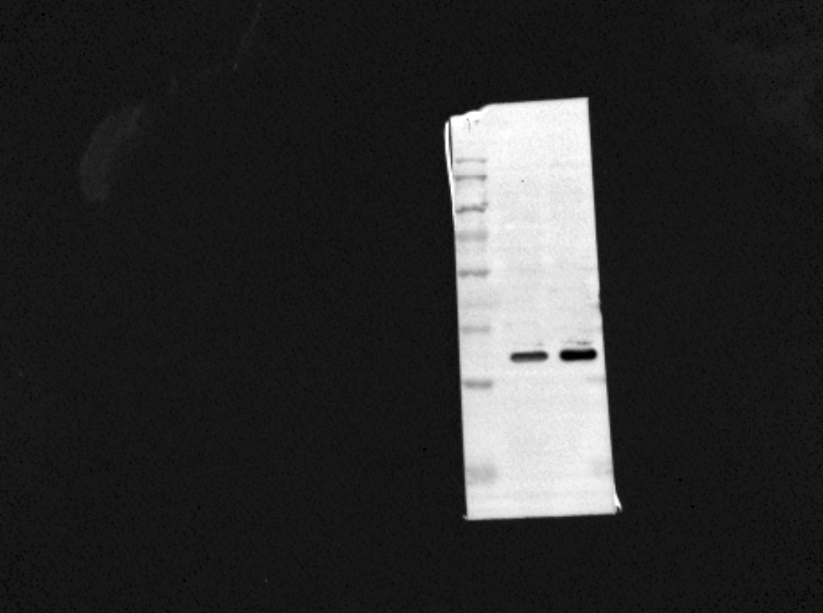


Fig. 4B input A549


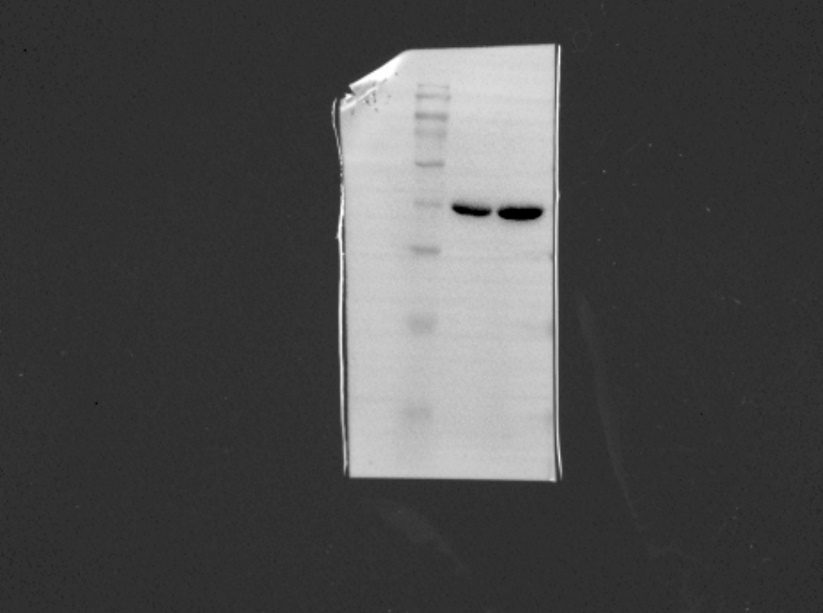


Fig. 4B input PC-9


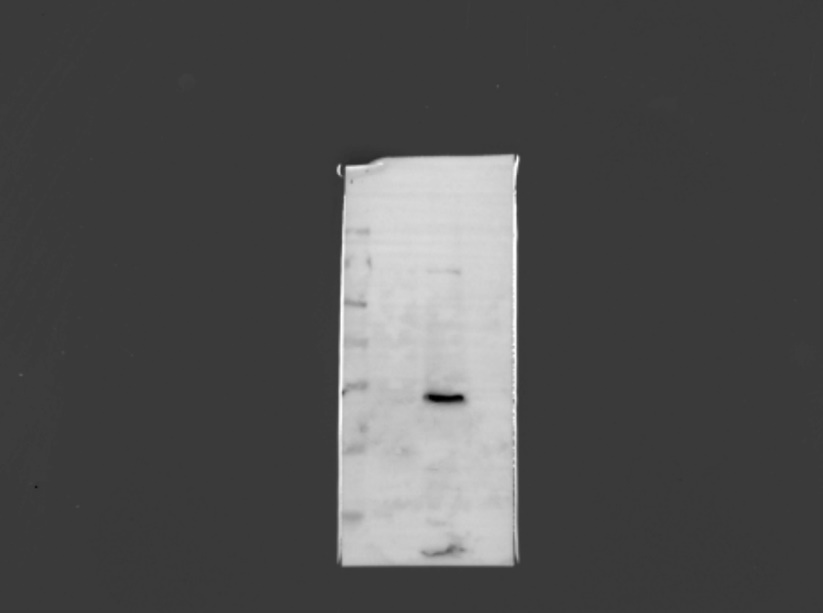


Fig. 4B IP A549


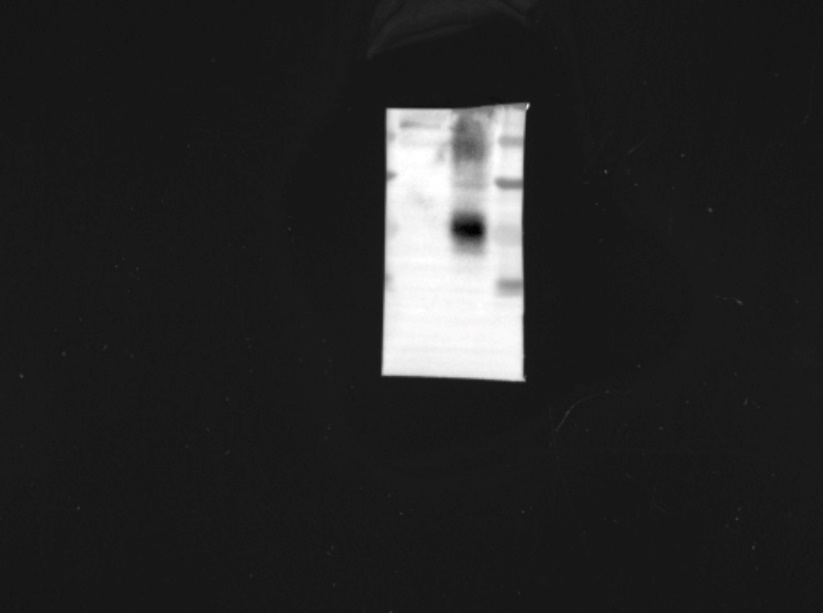


Fig. 4B IP PC-9
